# Machine learning-assisted analysis of epithelial mesenchymal transition pathway for prognostic stratification and immune infiltration assessment in ovarian cancer

**DOI:** 10.3389/fendo.2023.1196094

**Published:** 2023-06-19

**Authors:** Qian Li, Xiyun Xiao, Jing Feng, Ruixue Yan, Jie Xi

**Affiliations:** Department of Gynecology, Cangzhou Central Hospital, Cangzhou, Hebei, China

**Keywords:** serous ovarian cancer, epithelial mesenchymal transition, transcriptomics, single-cell sequencing, machine learning

## Abstract

**Background:**

Ovarian cancer is the most lethal gynaecological malignancy, and serous ovarian cancer (SOC) is one of the more important pathological subtypes. Previous studies have reported a significant association of epithelial tomesenchymal transition (EMT) with invasive metastasis and immune modulation of SOC, however, there is a lack of prognostic and immune infiltration biomarkers reported for SOC based on EMT.

**Methods:**

Gene expression data for ovarian cancer and corresponding patient clinical data were collected from the TCGA database and the GEO database, and cell type annotation and spatial expression analysis were performed on single cell sequencing data from the GEO database. To understand the cell type distribution of EMT-related genes in SOC single-cell data and the enrichment relationships of biological pathways and tumour functions. In addition, GO functional annotation analysis and KEGG pathway enrichment analysis were performed on mRNAs predominantly expressed with EMT to predict the biological function of EMT in ovarian cancer. The major differential genes of EMT were screened to construct a prognostic risk prediction model for SOC patients. Data from 173 SOC patient samples obtained from the GSE53963 database were used to validate the prognostic risk prediction model for ovarian cancer. Here we also analysed the direct association between SOC immune infiltration and immune cell modulation and EMT risk score. and calculate drug sensitivity scores in the GDSC database.In addition, we assessed the specific relationship between GAS1 gene and SOC cell lines.

**Results:**

Single cell transcriptome analysis in the GEO database annotated the major cell types of SOC samples, including: T cell, Myeloid, Epithelial cell, Fibroblast, Endothelial cell, and Bcell. cellchat revealed several cell type interactions that were shown to be associated with EMT-mediated SOC invasion and metastasis. A prognostic stratification model for SOC was constructed based on EMT-related differential genes, and the Kapan-Meier test showed that this biomarker had significant prognostic stratification value for several independent SOC databases. The EMT risk score has good stratification and identification properties for drug sensitivity in the GDSC database.

**Conclusions:**

This study constructed a prognostic stratification biomarker based on EMT-related risk genes for immune infiltration mechanisms and drug sensitivity analysis studies in SOC. This lays the foundation for in-depth clinical studies on the role of EMT in immune regulation and related pathway alterations in SOC. It is also hoped to provide effective potential solutions for early diagnosis and clinical treatment of ovarian cancer.

## 1 Introduction

Ovarian cancer is a prevalent gynaecological malignancy that has a deleterious effect on women’s health, accounting for approximately 3% of gynaecological malignancies worldwide. The incidence of ovarian cancer is positively correlated with the Human Development Index, and developing countries, mainly China, are at risk of increasing ovarian cancer incidence, according to GLOBOCAN 2020 (1). Global cancer statistics show that approximately 310,000 new cases of ovarian cancer occur each year, and approximately 150,000 people die from ovarian cancer (2, 3). The pathological types of ovarian cancer are also complex, with serous ovarian cancer (SOC) being one of the more important pathological subtypes. The mortality rate of SOC is also decreasing year by year with the improvement of medical treatment and the rapid progress in the development of therapeutic drugs (4–6). Despite the current improvements in the diagnosis and treatment of SOC, the 5-year survival rate of ovarian cancer patients has not improved significantly (7, 8). This shows that accelerating the research on SOC can not only improve the survival rate of ovarian cancer patients, but also reduce the disease burden caused by female malignancies and advance the development of healthcare.

Tumour invasion and metastasis is a complex process involving multiple genes and steps, with weakened adhesion and enhanced movement between tumour cells as the basis for invasion and metastasis. The potential of tumour invasion and metastasis depends on the interaction of internal environmental factors, of which epithelial-mesenchymal transition (EMT) is one of the main factors (9). EMT has also been shown to be a major cause of invasion and metastasis in epithelial ovarian cancer (10, 11). Epithelial mesenchymal transition (EMT) is a process by which epithelial cells lose their polarity and adhesion and acquire a mesenchymal phenotype. This process results in reduced adhesion and increased motility between tumour cells, involves multiple signalling pathways (12), is a necessary initial step for tumour cell invasion and metastasis, and is associated with malignant transformation and the development of metastasis, recurrence and drug resistance in a variety of malignancies, including ovarian cancer (13). In ovarian cancer, there is a small population of ‘tumour initiating cells’, characterised as mesenchymal and stem cells, which play a role in driving tumour initiation (14). The presence of TGFβ, a stimulating factor that induces EMT in ovarian follicular fluid, has been shown to inhibit PAX2, which maintains the differentiation of tubal epithelial cells, leading to the progression of intraepithelial tubal carcinoma to high-grade plasma SOC (15). It has also been shown that BRCA1 mutations induce EMT and tumourigenesis, often developing into highly invasive, poorly differentiated plasma ovarian cancer (16). Therefore, EMT plays an important role in both the pathogenesis of SOC and its development and invasion throughout the metastatic process. However, there is a lack of specific mechanisms of EMT-mediated SOC invasion and metastasis as well as single-cell descriptions of EMT-related genes in SOC. In addition, the construction of EMT-based prognostic stratification biomarkers for SOC is also important for the assessment of the efficacy of immunotherapy and the selection of therapies. Unfortunately, there is a lack of reported studies related to the above.

To address the existing and potential mechanistic evidence of EMT in the development of SOC, this paper will analyse the relationship between EMT and infiltrative metastasis of ovarian cancer in the hope of providing new ideas for the treatment of SOC. In this study, cell type annotation and spatial expression analysis of single cell sequencing data from the GEO database were performed using ovarian cancer gene expression data collected from the TCGA database and the GEO database and corresponding patient clinical data. To understand the cell type distribution of EMT-related genes in SOC single-cell data and the enrichment relationship between biological pathways and tumour function. Major differential genes for EMT were screened and a prognostic risk prediction model for SOC patients was constructed. We then used data from 173 SOC patient samples obtained from the GSE53963 database for the validation of the prognostic risk prediction model for ovarian cancer. In addition, GO functional annotation analysis and KEGG pathway enrichment analysis were performed on mRNAs predominantly expressed with EMT to predict the biological function of EMT in ovarian cancer. We also analysed the direct association of SOC immune infiltration and immune cell regulation with EMT risk scores, laying the foundation for in-depth clinical studies of EMT in SOC immune regulation and related pathway alterations. This study further explores the molecular mechanism of ovarian cancer metastasis and to provide potential markers and therapeutic targets for the diagnosis of early cancer metastasis in ovarian cancer.

## 2 Materials and methods

### 2.1 Data acquisition

Biological data refers to the measurement and collection of different kinds of genomic, transcriptomic, epigenomic, proteomic and metabolomic data of organisms by modern sequencing techniques as well as histological techniques. The main biological databases used in this paper include, gene expression databases (TCGA database, GEO database), single cell sequencing databases (GEO database) and gene enrichment analysis databases (GO database, KEGG database). The data for this study were obtained from The Cancer Genome Atlas (TCGA) database and individual clinical information data. The ovarian cancer mRNA gene data used in this paper were obtained from ovarian cancer gene expression data obtained from the TCGA database. Samples of the original RNA-Seq format ovarian cancer sequencing data were downloaded from this database with complete gene sequencing results and complete clinical information on the patients. In addition, bulk RNA-seq of 174 ovarian cancer patients from GSE53963, and single-cell scRNA-seq data of 5 high-grade SOC patients from GSE154600 were included for further analysis.

### 2.2 Single-cell sequencing annotation of SOC and spatial distribution profile of EMT-related markers

Single-cell transcriptome sequencing refers to the high-throughput sequencing of mRNAs after reverse transcriptional amplification at the individual cell level. By sequencing at the single cell level, single cell sequencing solves the problem of not being able to obtain information about the heterogeneity of different cells with tissue samples or having too small a sample size for routine sequencing, and provides a new direction for scientists to study the behaviour and mechanisms of individual cells. Due to cellular heterogeneity, the genetic information of cells of the same phenotype may differ significantly, and much of the low abundance information will be lost in the overall characterization. In order to compensate for the limitations of traditional high-throughput sequencing, single-cell sequencing has been developed. The basic steps of single cell sequencing: isolation of selected single cells → amplification → high throughput sequencing → data analysis using bioinformatics techniques. In this study, we accessed the NCBI GEO website: https://www.ncbi.nlm.nih.gov/geo/ to download the data. The SOC-related single cell data were searched and downloaded from the search box. First, single-cell analysis was performed on five high-grade SOC samples from the scRNA-seq data of GSE154600, downscaled to six cell populations. t-SNE’s main use is to visualise and explore high-dimensional data. It was developed and published by Laurens van der Maatens and Geoffrey Hinton in JMLR Volume 9 (2008). The main goal of t-SNE is to transform multidimensional datasets into low-dimensional datasets. Compared to other dimensionality reduction algorithms, t-SNE works best for data visualisation. If we apply t-SNE to n-dimensional data, it will intelligently map n-dimensional data to 3d or even 2d data and the relative similarity of the original data is very good. Like PCA, t-SNE is not a linear dimensionality reduction technique, it follows non-linearity, which is the main reason why it can capture the complex flow structure of high-dimensional data. The main regions of the spatial distribution of single cells from five LIHC patients are depicted, thus providing insight into the expression of the main SOC tumour cell types and their relative proportions. In addition, we present a map of the spatial distribution of single cell data from five SOC patients. to understand the individual differences in cell annotation across patients. To understand the cell types and specific functional modalities of the major EMT roles in SOC, the spatial distribution of expression in the annotated major cell types was annotated using a variety of specific markers of EMT that have been widely formalised. The main EMT markers included were: EPCAM, LUM, RAMP2, COL3A1, CD79A, CD3D, CD8A, LYZ, CD68. We also compared and analysed the percentage and distribution levels of the different cell types in single cell samples from each SOC patient.

### 2.3 Signal communication and biological function enrichment analysis of SOC major annotated cell types

After initial pre-processing and downscaling analysis by scRNA-seq of GSE154600, we sought to understand the interactions between different cell types and signalling guidance. This is to better understand the microscopic role of EMT in the development of SOC. The intercellular communication network (ICN) is a weighted directed graph consisting of significant ligand-receptor pairs between interacting cell groups, showing the number of detected ligand-receptor interactions between different cell groups. cellChat uses the out-degree, in degree to infer the strength of different cell groups as senders, and receivers of signals during cellular communication. To further analyse intercellular communication in a more biologically meaningful way, ligand-receptor pairs are grouped into functionally relevant signalling pathways and CellChat is able to quantify the similarity between all significant signalling pathways, grouping them according to the similarity of their cellular communication networks. Pattern recognition was used to predict coordinated responses between cells. Therefore, we performed interaction resolution of single cell data from SOC by cellchat analysis. In addition, we calculated GSVA enrichment pathway scores for six cell populations using 50 Hallmark datasets.GSVA gene set variation analysis, an analysis of microarray and RNA-seq data gene sets under parameter-free and unsupervised conditions.GSVA enables the enrichment of a gene - sample data matrix (GSVA converts a gene-sample data matrix (microarray data, FPKM, RPKM, etc.) into a gene-set-sample matrix. Based on this matrix, the enrichment of gene sets (e.g. KEGG pathway) in individual samples can be further analysed. As GSVA results in a gene-set-sample enrichment matrix, there is more freedom for downstream analysis than with other gene-set enrichment methods.

The GSEA database of EMT (Epithelial Mesenchymal Transition) pathways was scored for gene set enrichment using the AddModuleScore function of the Seruat package and divided into two groups: EMT-high level and EMT-low level. GO analysis, KEGG analysis and GSEA-GO were performed using the R package “clusterProfiler” (17)(version 4.0.5), with a false discovery rate (FDR) < 0.05 to determine significant enrichment. Gene Onto Logy (GO) is a public database built by the Gene Onto Logy Consortium, which contains annotated information on the properties of species genes and related products, with the aim of standardising annotation information on the function of biological gene products. The Kyoto Encyclopedia of Genes and Genomes (KEGG), developed by the Kanehisa Laboratory in collaboration with the Institute of Chemistry of Kyoto University, integrates information on genomes, biological pathways, chemicals and system functions from the Human Genome Project. KEGG is a systematic knowledge base that uses specific algorithms to access and collate the results of existing experiments. The database can be divided into four main modules: Systems Information, Genomic Information, Chemical Information and Health Information. The KEGG Pathway database in the Systems Information module is a standard biological pathway database that is highly recognised in the field of bioinformatics research and can be used to explain biological processes within cells in a graphical form.

### 2.4 Construction of prognostic biomarkers for SOC based on EMT risk genes and functional validation of the model

The original dataset of RNAseq data in HTSeq-Counts format was converted to TPM format followed by Log2 transformation. First, we pre-processed the transformed data using the R language survival package and did one-to-one one-way Cox analysis of the EMT-associated gene datasets with significant expression differences. Secondly, as the traditional Cox regression model is only applicable when the number of covariates is smaller than the number of samples, when the number of covariates is larger than the number of samples, the model parameters will be difficult to be calculated. Also, there may be a high degree of similarity in gene expression. To reduce the appearance of overfitting of the results, we took Least absolute shrinkage and selection operator (LASSO) downscaling of the EMT-related genes that regressed significantly after the one-way Cox regression to screen out the EMT-related genes that were more correlated with survival outcomes. The major genes obtained by LASSO regression were combined with their linear coefficients to form an EMT-related risk score, and the median risk score differentiated the Lowrisk and Highrisk groups to establish a Riskscore score. The Kaplan-Meier survival curve analysis was used to further analyse the survival of the EMT risk scores obtained from the LASSO regressions in relation to their corresponding clinical survival times of patients. A forest plot was used to calculate the proportion of risk for each EMT-related gene included in the model. Box plots were also used to depict the mRNA expression of EMT-associated genes in patients in the low-risk versus high-risk groups of the EMT score. In this paper, EMT-related genes obtained from screening in the Cox risk assessment model and m RNAs that were differentially expressed in ovarian cancer and normal ovarian tissues were selected and put into a co-expression network for analysis. The Pearson correlation coefficients between two different EMT genes included in the model were calculated based on the gene expression values. In addition, a mutational demonstration of the TCGA-OV cohort was performed. The mutation data were visualised by using the R package “maftools” (version 2.12.0).

### 2.5 Immune infiltration association analysis and drug therapy sensitivity assessment of EMT risk model

There is an increasing emphasis on immunotherapy in the field of oncology treatment, and infiltrating immune cells in the tumour microenvironment are an important cellular component and have a potential role in relation to tumour cells that may influence tumour progression and patient prognosis survival. To fully understand whether key EMT genes are associated with immune activity, this paper uses the R software GSVA (18) package to assess the expression levels of target EMT genes in relation to the infiltration of immune cells in tumour tissue. The Single Sample Gene Set Enrichment AnalysisA (ss GSEA) algorithm is based on the principle of calculating an enrichment score for a given gene set for each sample. The ss GSEA in immune infiltration uses markers specific to each type of immune cell as a gene set to calculate an enrichment score for each type of immune cell in each sample, inferring the infiltration of immune cells in each sample. the Xcell algorithm allows conversion of gene expression profiles to enrichment scores for 64 immune and stromal cell types across samples. Differences in the composition of cell types across subjects can identify cellular targets of disease and suggest novel therapeutic strategies. Therefore, we calculated immune infiltration scores using 2 methods: ssGSEA, xCell algorithm, visualised with box line plots, heat maps and scatter plots, respectively. ssGSEA calculates enrichment scores for single samples and gene set pairs to determine the degree of immune infiltration. xCell quantifies the abundance of 67 immune cells using transcriptomic data. In addition, adjusting for these variants allows detection of true gene expression differences and improves interpretation of downstream analyses.

To clarify the relationship between EMT immune infiltration and tumour immunotherapy and drug sensitivity, we further evaluated the efficacy of EMT risk scores stratified with tumour drug resistance and multiple drug therapy sensitivity phenotypes. Sensitivity scores were calculated for drugs in the GDSC database based on the R package “oncoPredict”, thus extending the clinical application of the EMT risk score.

### 2.6 GAS1 gene in epithelial mesenchymal transition regulates the development of SOC invasion - experimental validation at the cellular level

Firstly, we collected clinical samples of ovarian cancer by collecting 10 tumour samples and 10 paracancerous tissues. The qPCR technique was used to obtain the transcript expression of GAS1 gene in tumour tissues and normal tissues. qPCR experiments were performed in a similar way as reported in previous studies.The further ovarian cancer cell level experiments were set up as follows.

#### 2.6.1 Cell culture and gas1 knockdown validation

SKOV3 as well as 3AO cell lines were obtained from the ATCC repository. All 2 cell lines were cultured in McCoy’s 5a medium supplemented with 10% fetal bovine serum (FBS). (GIBCO, Thermo Fisher, Carlsbad, CA) and 1% antibiotic-antimycotic (#15240062, Thermo Fisher). All cell lines were stored in a humidified incubator at 37°C containing 5% CO2. GAS1 stable knockdown clones of both cell lines were obtained by using Mission shRNA (knockdown [KD]1: TRCN0000084044, KD2: TRCN0000294078, Sigma). MISSION(R) pLKO.1-puro empty vector (SHC001, Sigma) was used as shRNA control (Ctl). For lentivirus generation, HEK293T cells were transfected with 4.05 µg of target plasmid, 0.45 µg of pCMV-VSV-G (#8584, Addgene) and 3.5 µg of pCMV delta R8.2 (#12263, Addgene) using Lipofectamine 2000 (Invitrogen) for 24 hours. Cells were incubated with virus-containing supernatant and 8 ug/mL of polyethylene glycol for 24 hours. Purimycin was screened for 2 weeks. For tail vein injection, GAS1 shCtl or knockout SKOV3 as well as 3AO cells were stably transduced with a lentiviral vector. Transduction was performed with a lentiviral vector carrying red fluorescent protein (RFP), followed by fluorescence-activated cell sorting (FACSAria II, BD Biosciences, San Jose, CA) and expanded *in vitro*. WB experiments were performed to analyse the transcriptional expression of GAS1 in control, SI-control, and SI-GAS1 groups.

#### 2.6.2 CCK-8 test

Logarithmic growth stage cells were taken, digested with 0.25% trypsin, centrifuged, diluted into single cell suspensions with complete medium containing 10% fetal bovine serum, inoculated into 96-well plates, adjusted to 8 000 cells/well, incubated at 37 °C in a 5% CO2 incubator, and 20 μL of CCK8 solution was added to each well at 24 h, 48 h, 72 h and 96 h. After 2 h, the absorbance (A) value at 450 nm was measured by enzyme marker. The absorbance (A) values at 450 nm were measured on an enzyme marker after 2 h.

#### 2.6.3 Colony formation assay

To assess colony formation, 100 shCtl or GAS1 knockdown cells were plated in triplicate on 6-well plates. 15 days later, cells were fixed in methanol for 2 min and stained with 0.2% (W/V) crystal violet for 30 min. Colonies were counted using GelCount™ (Oxford Optoronix, Abingdon, UK) and the values were normalised to the control.

#### 2.6.4 Transwell experiment

Matrix gel was used to spread the plates, cell suspensions were made, walled cells were digested using trypsin, washed with PBS and then inoculated cells were resuspended in serum-free medium, the cell suspensions were added to Transwell chambers, incubated for 24 h and fixed, and finally stained and counted. Control, SI-control and SI-GAS1 invasion were analysed.

### 2.7 Statistical analysis

Statistical analysis was performed using R tools and GraphPad Prism8. Data were expressed as mean ± SD (standard deviation) from 3 independent measurements. Each experiment was repeated 3 times, and the P < 0.05 means statistically significant.

## 3 Results

### 3.1 Annotated analysis and downscaling clustering of single cell samples from GSE154600

**Figure 1:**
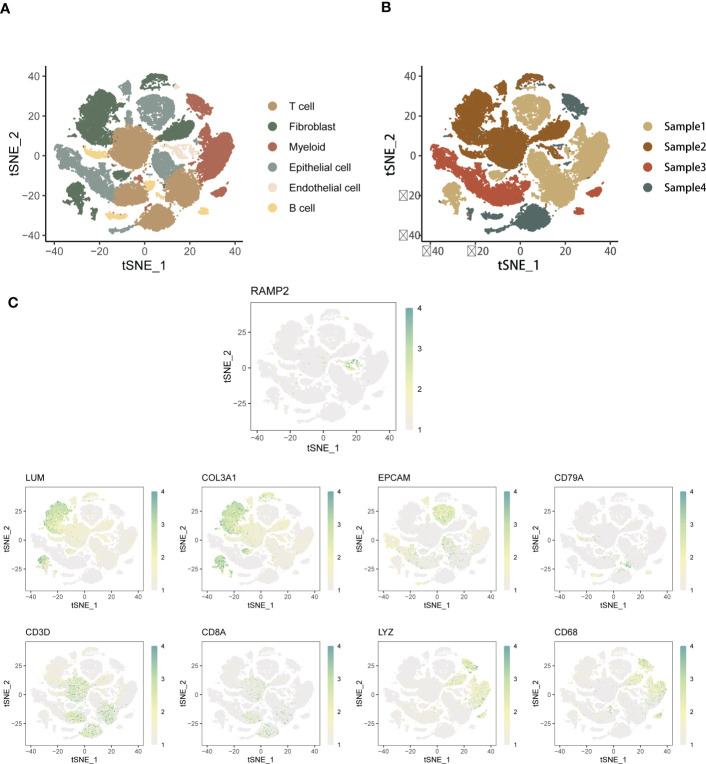
Annotated analysis and descending clusters of single cell samples from GSE154600. **(A)** scRNA-seq analysis of 4 soc samples from GSE154600, t-sne revealed cell clustering including Hepatocyte, T cell, Myeloid, Fibroblast, Epithelial Cell, Endothelial cell, and B cell; **(B)** Single cell sequencing Annotated maps of spatial distribution were obtained for four SOC patients; **(C)** Annotated maps of the spatial distribution of expression in annotated SOC cell types by widely formalized EMT-specific markers. The main EMT markers included were: EPCAM, LUM, RAMP2, COL3A1, CD79A, CD3D, CD8A, LYZ, CD68 (blue-green represents high expression, white represents low expression).

First, we performed cell type annotation analysis and downlink clustering on four single-cell samples from the GSE154600 database. Major cell clustering and downlinked expression analysis were performed using the t-sne method. First, we performed a generalized analysis of the major cell types in the obtained SOC single-cell samples and color differentiated them according to the differences in the obtained cell types. Six main cell clusters were identified and labeled in the samples, and the major cell clusters were labeled in the form of (**Figure 1A**):T cell, Myeloid, Fibroblast, Epithelial Cell, Endothelial cell, and B cell. there were some differences in the expression distribution and content percentage of each cell cluster, but all of them had significant expression in the SOC single-cell The expression distribution and content of each cell group differed somewhat, but all of them were significantly expressed in the SOC single cell samples. T cells, Fibroblast and Myeloid were the main cells expressed in SOC, except for Endothelial and B cells, which were relatively less expressed. Further, we spatially annotated the respective spatial distribution as well as cell expression correlations of the four samples (**Figure 1B**). The results showed that Epithelial, Myeloid as well as T cells were predominantly expressed in case 1, Fibroblast and T cells were predominantly present in case 2, Epithelial and T cells were the predominantly expressed cells in patient 3, and the predominantly expressed cells in patient 4 were proximal to those in patient 3. EMT-specific markers that have been widely formalized annotate the spatial distribution of expression in the annotated SOC cell types. The main EMT markers included: EPCAM, LUM, RAMP2, COL3A1, CD79A, CD3D, CD8A, LYZ, CD68 (**Figure 1C**). expression of EPCAM overlaps with Epithelial cell enrichment, LUM is mainly located in patient 2 and Fibroblast, T cell expression regions, RAMP COL3A1 was present in a variety of cell types, including Fibroblast, T cells, etc. CD79A was present in SOC samples as a marker for B cells. CD3C, CD8A were expressed in the T cell region. CD68 were mainly associated with Meloid distribution. In **Figure S1A**, we performed a histogram analysis of the percentage of major cell types for the four SOC samples. For sample 1, T cells, Myeloid, and Epithelial were the predominant cell types. Myeloid expression was lacking in sample 2, and the relative expression was similar in samples 3 and 4, with Epithelial, and T cells occupying a larger expression.

### 3.2 Signaling communication and pathway enrichment analysis of major annotated cell types of EMT-related SOC

The above study gave us a preliminary understanding of the major cell types and annotation of SOC single cell samples, with immune cells occupying the major cell expression and functional distribution weights. Therefore, we further describe the SOC tumor pathways and biological functions associated with the analysis of EMT expression. Fibroblast is the most active and interacting cell type in SOC samples, and its signals are mainly directed to Myeloid and Endothelial cells. The communication from Endothelial cells was mainly focused on Myeloid, while less signals from B cells occurred in Epithelial. Further, GSVA scoring was used to reveal the correlation between the expression of different cell types and the main tumor pathways (**Figures S1C**, **D**). T cells positively correlated with ALLOGRAFT-REJECTION expression, and Fibroblast was mainly enriched in Epithelial mesenchymal transition. Myeloid is positively co-expressed with multiple functional pathways, including E2F_TARGETS, inflammatory response and fatty acid metabolism. B cells were mainly enriched in Epithelial mesenchymal transition. This shows a significant correlation between the differential expression of EMT in different cell types further demonstrating the relationship between the enrichment of tumor functional pathways.

**Figure 2:**
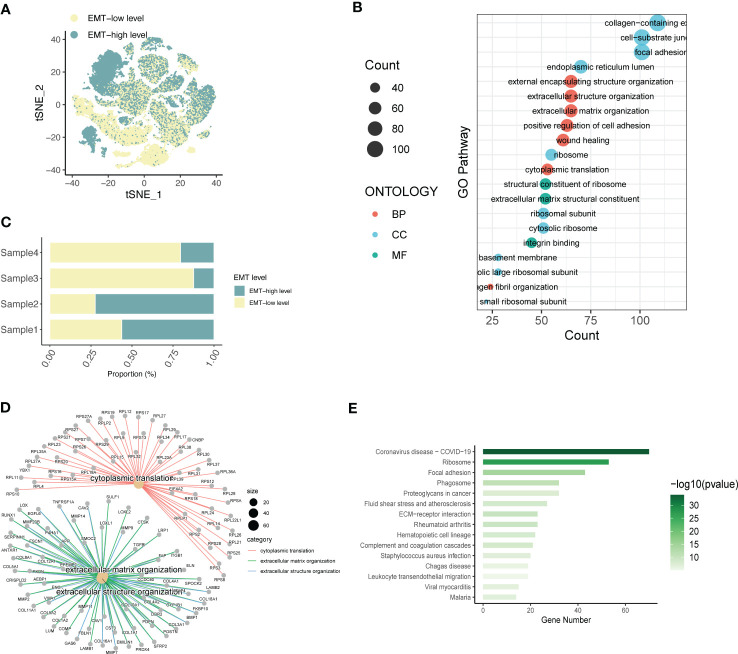
Expression annotation and biological pathway enrichment analysis of EMT-related genes. **(A)** Single-cell spatial distribution maps of EMT signature-high and EMT signature-low groups obtained from gene set enrichment scoring; **(B)** Percentage of EMT-high and EMT-Low expression in four SOC single-cell data samples; **(C)** GO analysis depicting the major enrichment pathways mediating tumor infiltration by the major acting cell types in LIHC and **(D)** Depiction of extracellular matrix network analysis; **(E)** Histogram of KEGG analysis showing the enrichment of tumour pathways associated with high EMT expression.

To understand the spatial distribution of expression of different SOC annotated cell types, we also used the AddModuleScore function of the Seruat package to score the gene set enrichment of the EMT pathway in the GSEA database into two groups, EMT-high level and EMT-low level. Spatial annotation was seen (**Figure 2A**), Fibroblast was mainly EMT-high level, T cell was mainly EMT-low level group, and other cells were a mixture of the 2 groups. It is speculated that this may be related to the role of different cell types in the EMT process of SOC. Fibroblasts are the main function-playing role of EMT, while T cells are involved in the anti-fibrotic process and tumor immune response, and thus have lower expression of related genes. In addition, we analyzed the EMT gene expression in four SOC patients using a hierarchical bar graph (**Figure 2B**). Patient 1 and patient 2 were predominantly EMT-high, while patient 3 and patient 4 had predominant EMT-Low occupant expression.

Based on this, the main functional pathways and biological mechanisms by which EMT plays a role in SOC are the next major thing we urgently need to understand, so we performed KEGG analysis and GO analysis on this basis. GO analysis revealed (**Figure 2C**) that the differential genes of ECM were mainly enriched in external encapsulating structure organization, extracellular structure organization, extracellular matrix organizatio, positive regulation of cell adhesion, wound healing; cell components are mainly enriched in collagen-containing, cell-substrate junction, focal adhesio, endoplasmic reticulum lumen, ribosome; molecular functional enrichment is expressed in structural constituent of ribosomal In addition, in **Figure 2D**, we also performed the analysis of extracellular matrix components and related gene expression. KEGG analysis further demonstrated the main relevant functions and enrichment pathways of EMT in SOC development. The main components include Ribosome,Focal adhesion,Phagosome,Proteoglycans in cancer,Fluid shear stress and atherosclerosis.

### 3.3 Construction of SOC prognostic biomarkers for EMT genetic risk model and functional assessment

**Figure 3:**
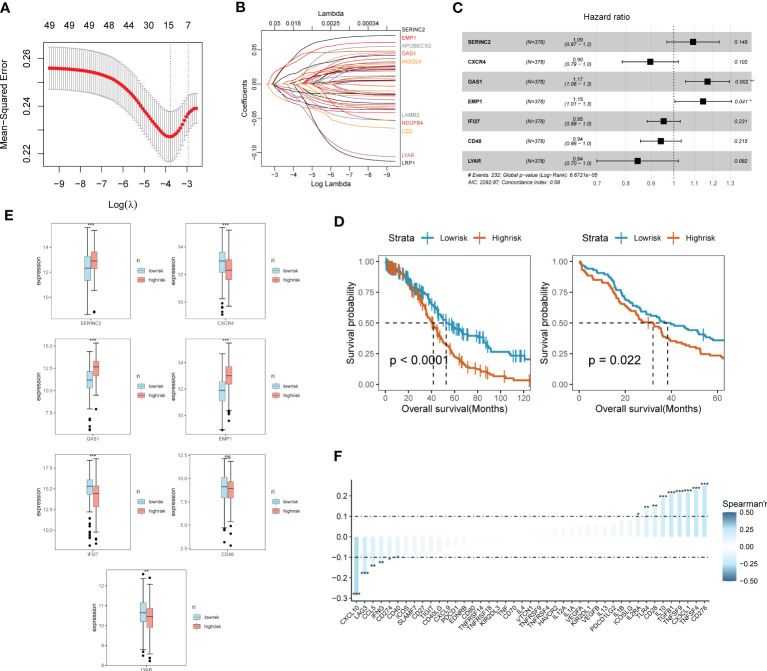
SOC survival model construction and functional validation of prognostic stratification for EMT risk markers. **(A)** Feature screening process curves from LASSO regression; **(B)** LASSO regression reveals feature screening of SOC risk model; **(C)** Forest plot demonstrating major EMT risk genes; **(D)** Prognostic value of EMT survival biomarkers assessed by survival curve Kaplan-Meier analysis in TCGA-OV training group and GEO validation; **(E)** Seven major EMT risk genes with EMT-HIGH and EMT-LOW expression box line plots; **(F)** Spearman expression correlation plots reveal the correlation between the expression of immune factors and EMT risk genes.

Through the previous studies on signaling communication and tumor pathway networks, we found that the major gene types of EMT may be closely associated with the development of SOC and invasive metastasis. Considering that there is a lack of EMT-related prognostic risk markers for SOC, we further analyzed the prognostic value of ECM-related genes for SOC. Key risk genes for the major differential genes of ECM were searched and prognostic models were composed. Using the TCGA-OV cohort as the training group, the cohort selected 7 genes of prognostic significance from 22 genes according to Lasso regression and constructed prognostic models. Multi-factorial Cox regression and LASSO analysis were performed on 50 prognosis-related EMT risk genes (**Figures 3A**, **B**), and 7 EMT risk genes with significant correlation with prognosis of ovarian cancer patients were obtained (P < 0.01), which constituted a prognostic prediction model for ovarian cancer. Meanwhile, the Risk Score (RS) of each patient was calculated in this paper. Then, patients were divided into high-risk and low-risk groups according to the median of risk score, and the forest plot of risk score and risk genes was drawn (**Figure 3C**). Among them, SEINC2, GAS1 and EMP1 were significantly positively expressed genes, while several other risk genes were mainly negatively expressed. The formula was: risk score = SERINC2*0.08956304 - CXCR4 *0.109059452 + GAS1*0.15692187+ EMP1*0.13645030 - IFI27*0.04696752 - CD48*0.05994817 - LYAR*0.16842919. **Figure 3D** shows that both in the training group of TCGA-OV and in the independent validation group of 173 ovarian cancer patients in GSE53963. The grouping curves based on EMT risk marker expression demonstrated significant prognostic stratification and survival predictive power. In addition, we analyzed the expression of seven EMT risk genes individually, in the EMT-High and EMT-Low groups. All seven expressed genes possessed significant differences between the two groups (**Figure 3E**). In addition, we examined the influence of the above model genes on the clinical prognosis of ovarian cancer, and found that the expressions of SERINC2, GAS1 and EMP1 were negatively correlated with clinical outcome, while the expressions of LYAR, IFI27, CXCR4 and CD48 were positively correlated with clinical outcome (**Figure S2**). Using Spearman correlation analysis, we described the correlation between EMT risk score and the expression of multiple tumor pathways and immune factors (**Figure 3F**). CXCL10, LAG3, CCL5, IFNG, CD274, and CD40 showed a significant negative correlation with EMT score expression.CD276, TNFSF4, CX3CL1, TNFSF9, and TGFB1, on the other hand, showed a significant positive correlation with the expression of EMT risk genes.

**Figure 4:**
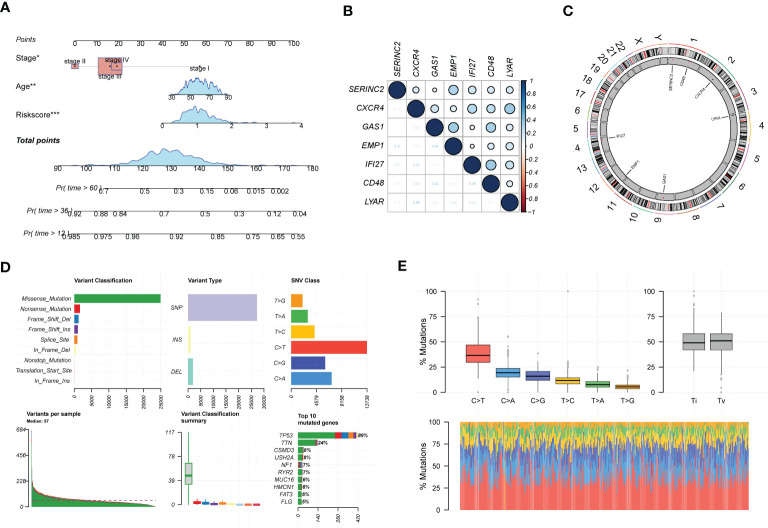
In-depth profiling and mutation analysis of EMT risk biomarkers. **(A)** Prognostic prediction exhibition of SOC risk markers consisting of age, Stage, and EMT Riskscore; **(B)** Correlation heat map demonstrating the expression correlation of seven EMT risk genes; **(C)** Chromosome distribution map revealing the chromosomal expression locations of seven EMT genes; **(D)** Chromosomal mutations and gene mutations depicted; **(E)** Base pair mutations and box line plot of mutation probabilities. *p < 0.05, **p < 0.01, ***p < 0.001.

In addition, we analyzed clinical indicators and risk scores by multifactorial COX regression and established an integrated model based on EMT. to meet the practical decision-making needs of clinical visibility and multifactorial integrated analysis. The final model consisting of Stage, age, and Riskscore was finally screened by COX analysis and incorporated, as shown in **Figure 4A**. **Figure 4B** shows the main expression correlations of the 7 EMT risk genes. positive correlation of SERINC2, EMP1 expression is strong. high correlation of CXCR4, IF127, LYAR expression. gas1 is mainly correlated with EMP1 and CD48. We also analyzed the main locations of the major genes in the EMT markers on human chromosomes (**Figure 4C**). SERINC2 and CD48 were located on chromosome 1, CXCR4 on chromosome 2, LYAR on chromosome 4, RAC1 on chromosome 7, and GAS1 on chromosome 9.EMP1 and IF127 were located on chromosomes 12 and 14, respectively. In addition, we also describe the mutations in the chromosomes of EMT-related genes in **Figures 4D**, **E**. Missense mutations and NONSENSE mutations are the major mutation forms. And the main mutated genes were TP53 and TTN.

### 3.4 Association assessment of SOC immune infiltration with EMT model expression

**Figure 5:**
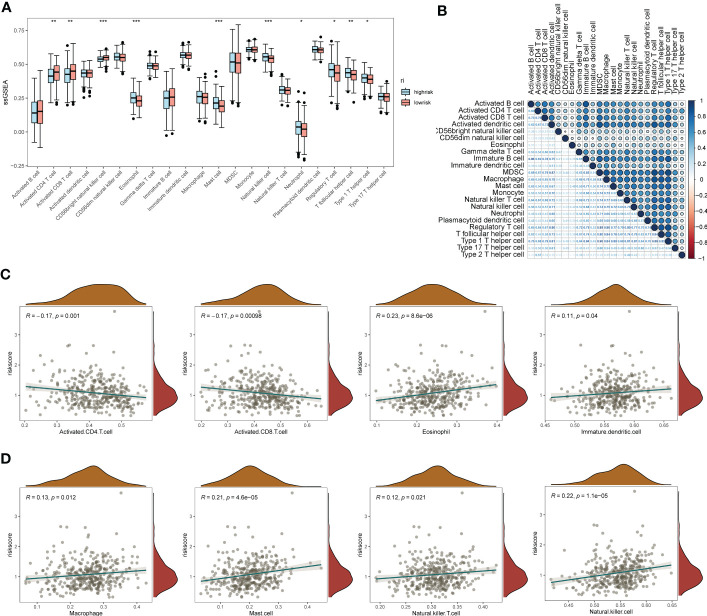
ssGSEA immuno-infiltration analysis of the EMT risk gene model. **(A)** Box line plot of ssGSEA revealing the differential box line plot of expression of major immune cells and immune pathways in the low and high risk groups for EMT; **(B)** Correlation heat map revealing the correlation of expression of major immune cells; **(C)** Scatter plot of linear correlation of expression of several major immune cells with EMT risk score; **(D)** Scatter plot of linear correlation of expression of several additional major immune cells with EMT risk model. *p < 0.05, **p < 0.01, ***p < 0.001.

The results of gene pathway enrichment and immunological analyses initially indicated that the regulatory role of EMT in SOC showed significant correlation mainly with immune pathway-related expression. Therefore, deeper immune infiltration analysis was used to characterize the impact of EMT-related risk genes in SOC in association with immune infiltration and regulation of immune cell expression. We chose the ssGSEA, xCell algorithm to calculate the immune infiltration score and visualize it in multiple forms. First of all, in the immune cell enrichment score calculated by ssGSEA we found (**Figure 5A**). Eosinophil, Mast cells, NK cells, T follicular helper cells, and Th1 possessed significant expression in the EMT high risk group. In **Figure 5B**, we calculated the correlation between the distribution of multiple tumor immune cells and found that the tumor distribution of multiple immune cells possessed significant specificity-related characteristics. To describe the direct association of EMT with tumor immune infiltration, we calculated and depicted the correlation between EMT risk score and immune cell expression by linear correlation scatter plots (**Figures 5C**, **D**). The results showed that activated CD4 T cells, activated CD8 T cells possessed a significant negative linear correlation with EMT risk score. eosinophil, Immature dendritic cells, Mast Cell, macrophages, NK cells and NK T cells showed a positive correlation with risk score expression. In **Figures S3A**, **S3B**, we also depicted the gene mutations in the high-risk and low-risk groups by chromosome analysis. TP53, TTN, and CSMD3 were the major mutation types in both the high- and low-risk EMT groups. XCell method further demonstrated the expression correlation between EMT risk grouping and tumor immune infiltration (**Figures S3C**, **S3D**).

**Figure 6:**
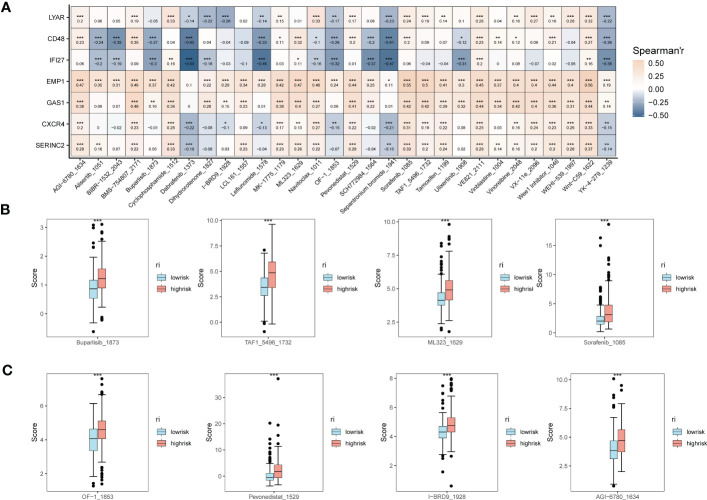
Expression correlation assessment of tumor treatment sensitivity and EMT risk score. **(A)** Correlation heat map demonstrating the therapeutic sensitivity of seven EMT risk genes with multiple chemotherapeutic agents; **(B)** Box plots of therapeutic sensitivity of Buparlisib_1873, Sorafenib_1085, TAF1_5496_1732, ML323_1629 in EMT-HIGH and EMT-LOW; **(C)** AGl-6780_ 1634, I-BRD9_1928, Pevonedistat_1529, OF-1_1853 box-line plots of treatment sensitivity in EMT-HIGH and EMT-LOW. *p < 0.05, **p < 0.01, ***p < 0.001.

In addition, to understand the correlation between the expression of EMT risk genes and the efficacy of tumor immunotherapy therapies, we also calculated sensitivity scores for the drugs in the GDSC database based on the R package “oncoPredict”. **Figure 6A** shows the correlation between EMT risk genes and the sensitivity of various immunotherapeutic drugs. In **Figures 6B**, **C**, we further clarify the correlation between the expression of EMT risk genes and treatment sensitivity, TAF1_5496_1732, and ML323_1629 all had higher treatment sensitivity scores in the high-risk group than in patients in the low-risk group for EMT. AGl-6780_1634, I-BRD9_1928, Pevonedistat_1529, and OF-1_1853 also had better treatment outcomes in the high-risk group for EMT.

### 3.5 GAS1 in epithelial mesenchymal transition regulates the development of SOC invasion - experimental validation at the cellular level

**Figure 7:**
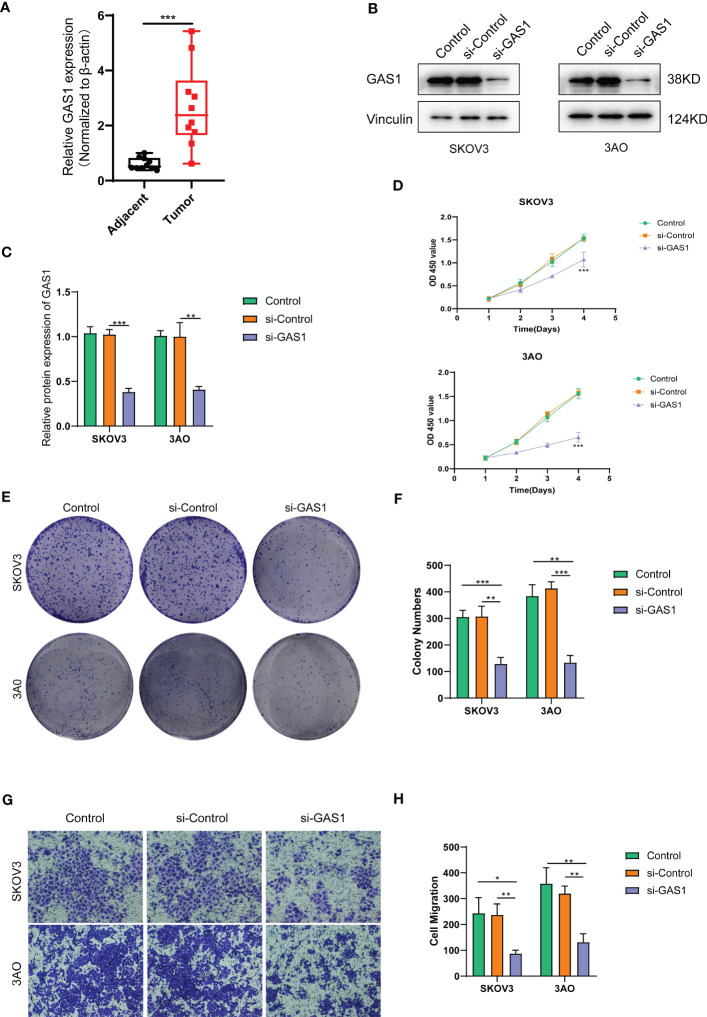
SOC validation of GAS1 gene regulation in epithelial mesenchymal transition at the cellular level. **(A)**. qPCR analysis of GAS1 expression histograms in 10 pairs of SOC tumor and paracancer samples; **(B)** WB bands demonstrating GAS1 gene expression in control, SI-control, and SI-GAS1; **(C)** Bar and bar graphs quantifying GAS1 gene expression in control, SI-control, and SI-GAS1; **(D)** CCK8 experiments revealed cell survival of two SOC cell lines in culture for 24h, 48h, 72h and 96h in control, SI-control and SI-GAS1; **(E)** Colony formation in control, SI-control, and SI-GAS1 demonstrated; **(F)** Control, SI-control, and SI- Colony formation histogram analysis of GAS1; **(G)** Transwell invasion assay comparing the effects of control, SI-control, and SI-GAS1 in two SOC cell lines; **(H)** Histogram analysis of Transwell assay for control, SI-control, and SI-GAS1. *p < 0.05, **p < 0.01, ***p < 0.001.

For the obtained tissue samples from ovarian cancer and paracancer, qPCR showed that GAS1 was significantly highly expressed in the tumour samples, with significant differences in GAS1 transcript expression between the two groups (**Figure 7A**). This firmly established the need for subsequent cellular experiments to clarify the specific effects of GAS1 on the regulation of invasion and metastasis in ovarian cancer cells.

**Figure 8:**
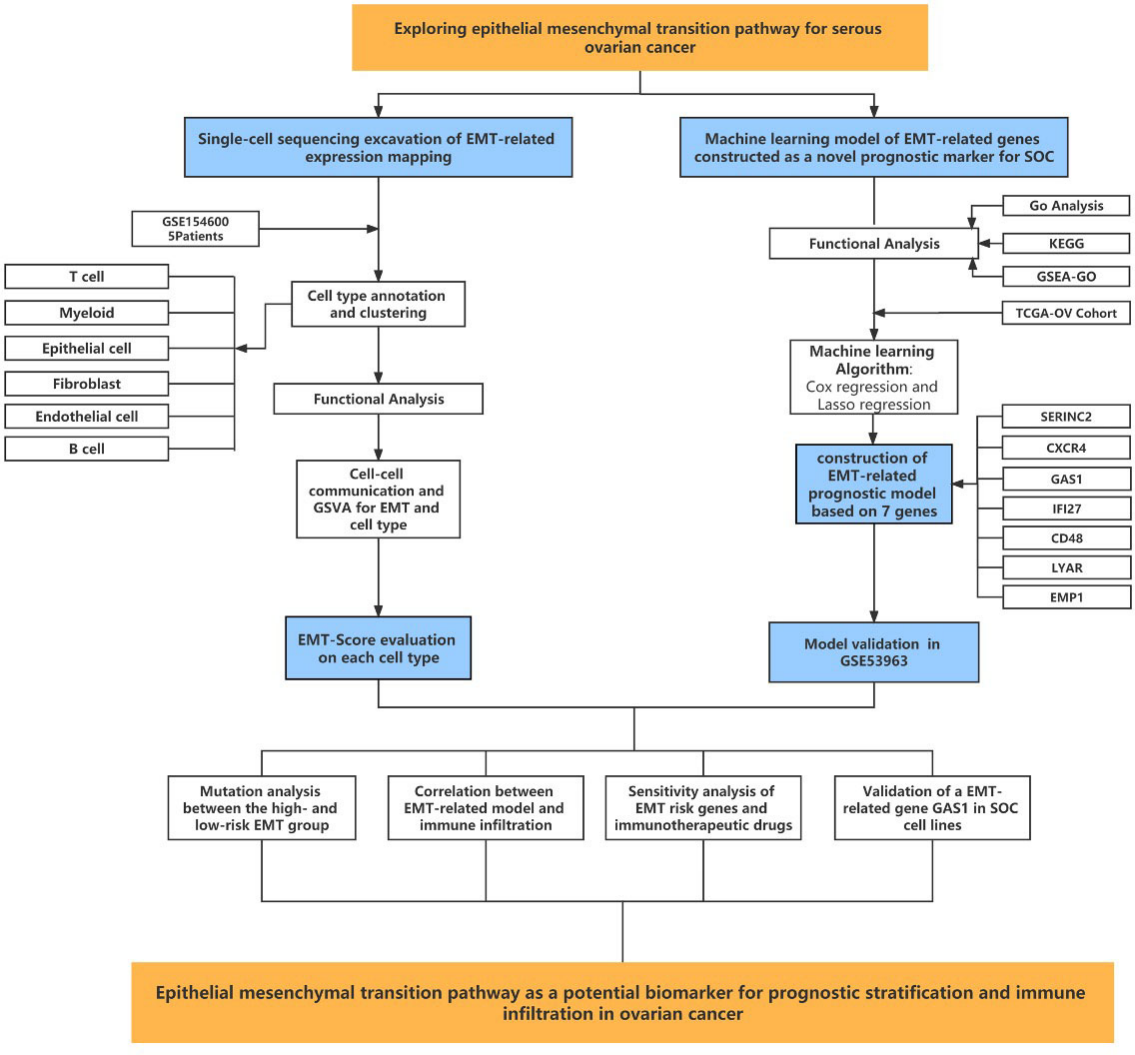
Flow chart showing the methodology of the study.

Subsequent cellular experiments were then carried out. We first verified the successful knockdown of the GAS1 gene in the strips by WB experiments. WB strips and WB quantitative analysis (**Figures 7B**, **C**) showed that GAS1 expression was significantly reduced in the SI-GAS1 group compared to the control and SI-control. On this basis, we assessed the effect of GAS1 gene affecting the proliferation of ovarian cancer cell lines by colony assay. **Figure 7D** shows the results of the CCK-8 experiments at different time periods for the cell survival rates of the two ovarian cancer cell lines. Contrast control, SI-control. The survival rate of ovarian cancer cells in the SI-GAS1 group decreased significantly as the culture time increased. Especially after 96h of culture, which suggests that GAS1 is a key gene for ovarian cancer cells to maintain viability. **Figure 7E** shows that pancreatic cancer cell colony formation was significantly reduced in the SI-GAS1 group. This suggests that genes associated with epithelial mesenchymal transition can regulate SOC aggregation and chemotaxis. Similarly, we also clarified this differential expression relationship using bar graphs (**Figure 7F**). Transwell assays showed that knockdown of the GAS1 gene significantly inhibited the proliferation, invasion and migration of ovarian cancer cells (**Figures 7G**, **H**). This suggests that epithelial mesenchymal transition and its associated genes profoundly influence the development of ovarian cancer. The flow chart of analyses was showed in **Figure 8**.

## 4 Discussions

Ovarian cancer has the highest mortality rate among all gynecologic malignancies and is on the rise. GLOBOCAN 2020 (19) showed 313,959 new cases of ovarian cancer and 207,252 new deaths worldwide in 2020, with 5-year survival rates below 40% (20) and mortality rates as high as 60%. Despite great progress in surgery and drug therapy, complete/partial remission is difficult to maintain in ovarian cancer patients, and exploring reliable biomarkers and precise molecular mechanisms is crucial for early diagnosis, treatment and prognosis of OC. In recent years, rapid advances in bioinformatics have enabled gene microarrays and sequencing data to provide a convenient and comprehensive platform for exploring general genetic alterations in tumors, identifying DEGs, and elucidating their molecular mechanisms for diagnosis, treatment, and prognosis (21, 22). Wu et al (23) found that Sialyltransferase ST3GAL1 promotes cell migration, invasion and TGF-β1-induced EMT, and confers ovarian cancer with paclitaxel resistance. Xu et al (24) revealed the four-EMT gene model used to predict the outcome of patients with HGSOC, the ability of mCAF to enhance the invasion of ovarian cancer cells, and the potential therapeutic value of anti-Tigit therapy through the transcriptome results of single cell sequencing analysis. Currently, biomarkers are not sufficiently studied by investigators and the results of DEGs are inconsistent; therefore, reanalysis of relevant database data may provide new ideas for current therapeutic studies in OC (25) to address the issues of prognosis, drug resistance, and recurrence of ovarian cancer. In this study, based on previous studies on the role of epithelial mesenchymal transition in SOC, EMT risk genes were identified and their spatial distribution was depicted by single-cell sequencing analysis. The main associations between EMT risk genes and tumor immunity were described by KEGG analysis and GO analysis. Meanwhile, the main functional role of EMT in SOC development was clarified by immune infiltration analysis and prognostic model construction. This lays the foundation for future construction of EMT-related risk markers and the study of their functional characteristics in the immune infiltration and pathway regulation of SOC.

Clinical work has revealed a high degree of heterogeneity in the growth, invasive metastasis, chemoresistance and other behaviors of ovarian cancer, suggesting that ovarian cancer is not a single disease but a group of diseases with different molecular phenotypes, pathogenesis and prognosis.In 2004, American pathologists proposed the doctrine of ovarian cancer dichotomy (26), which divided ovarian cancer into type I and type II ovarian cancer, followed by successive studies that found that the fallopian tube Subsequently, successive studies found the existence of lesions and precancerous lesions similar to high-grade plasmacytoma in the mucosa of the fallopian tubes, and therefore proposed the theory of tubal origin (27). Once the intraepithelial carcinoma of the fallopian tube is formed, the cells detach from the cilia to reach the ovarian surface and then form an invasive carcinoma. Migration of intraepithelial carcinoma cells from the fallopian tube to the ovary is a very important step in ovarian carcinogenesis. It has been shown that growth factors and hormones secreted by the ovary (28), such as TGFβ and activator A, have a role in inducing the migration of cancer cells to the ovarian surface. Activin A, which is released from the TGFβ superfamily in the follicular fluid during ovulation, can induce EMT and promote the migration of tubal epithelial cells and high-grade plasmacytoid ovarian cancer cells by activating PI3K/AKT and MEK/ERK pathways (29). In junctional plasmacytoid ovarian tumor cells, downregulation of p53 was found to promote the aggressiveness of junctional tumors by downregulating E-cadherin expression through the PI3K/AKT pathway (30). The main biological functions of EMT process in malignant tumors are to enhance cell motility and cellular drug resistance, and EMT has important research value in the development, clinical diagnosis and treatment of ovarian cancer. We hope to provide new reliable and specific therapeutic targets for ovarian cancer in the near future through in-depth study of EMT and key molecular nodes in the EMT-driven process. This also suggests the important role of constructing EMT-related risk markers for studying ovarian carcinogenesis and development.

The present study has several advantages and limitations. First, this paper is based on a multi-omics (single-cell sequencing data +Bulk sequencing data) model. No such systematic analysis has been conducted to explore the effect of EMT signaling pathway on ovarian cancer. while the prognostic model was constructed and validated using retrospective data from public databases, and more prospective data are needed to validate its clinical utility. Second, this study only included EMT-related models for prognostic modeling, which is difficult to avoid confounding factors, as there are many mutated prognostic genes in ovarian cancer that may be excluded. Further experiments will be conducted in the future to verify the relationship between EMT-related genes and tumor immunity.

Overall, this study constructed an EMT-based risk marker for SOC survival prediction and investigated its main functional characteristics and the exact association between it and tumor immune infiltration. In-depth study of the molecular mechanism of EMT can help to understand ovarian cancer more deeply. Enriching the mechanistic network of EMT in epithelial ovarian cancer will potentially identify potential therapeutic targets for the invasive and metastatic properties of epithelial ovarian cancer, which will help us create new drug targets and intervene in ovarian cancer and provide a more reliable basis for effective treatment of SOC.

## Data availability statement

The original contributions presented in the study are included in the article/**Supplementary Material**. Further inquiries can be directed to the corresponding author.

## Ethics statement

The studies involving human participants were reviewed and approved by Cangzhou Central Hospital. The patients/participants provided their written informed consent to participate in this study. Its number is LCYJ: NO. 2019-090.

## Author contributions

QL designed the study. XX, JF performed data analysis. RY drafted the manuscript. QL and JX revised the manuscript. All authors read and approved the final manuscript.
