# Supplementary material for: Machine learning-assisted analysis of epithelial mesenchymal transition pathway for prognostic stratification and immune infiltration assessment in ovarian cancer
Source: Front Endocrinol (Lausanne). 2023 Jun 19;14:1196094. doi: 10.3389/fendo.2023.1196094 (PMC10317337; doi:10.3389/fendo.2023.1196094)
Supplement: Supplementary file 1 [file Image_1.pdf]

Epithelial mesenchymal transition pathway as a potential biomarker for prognostic stratification and immune infiltration in ovarian cancer: a study based on single-cell sequencing and transcriptomic analysis

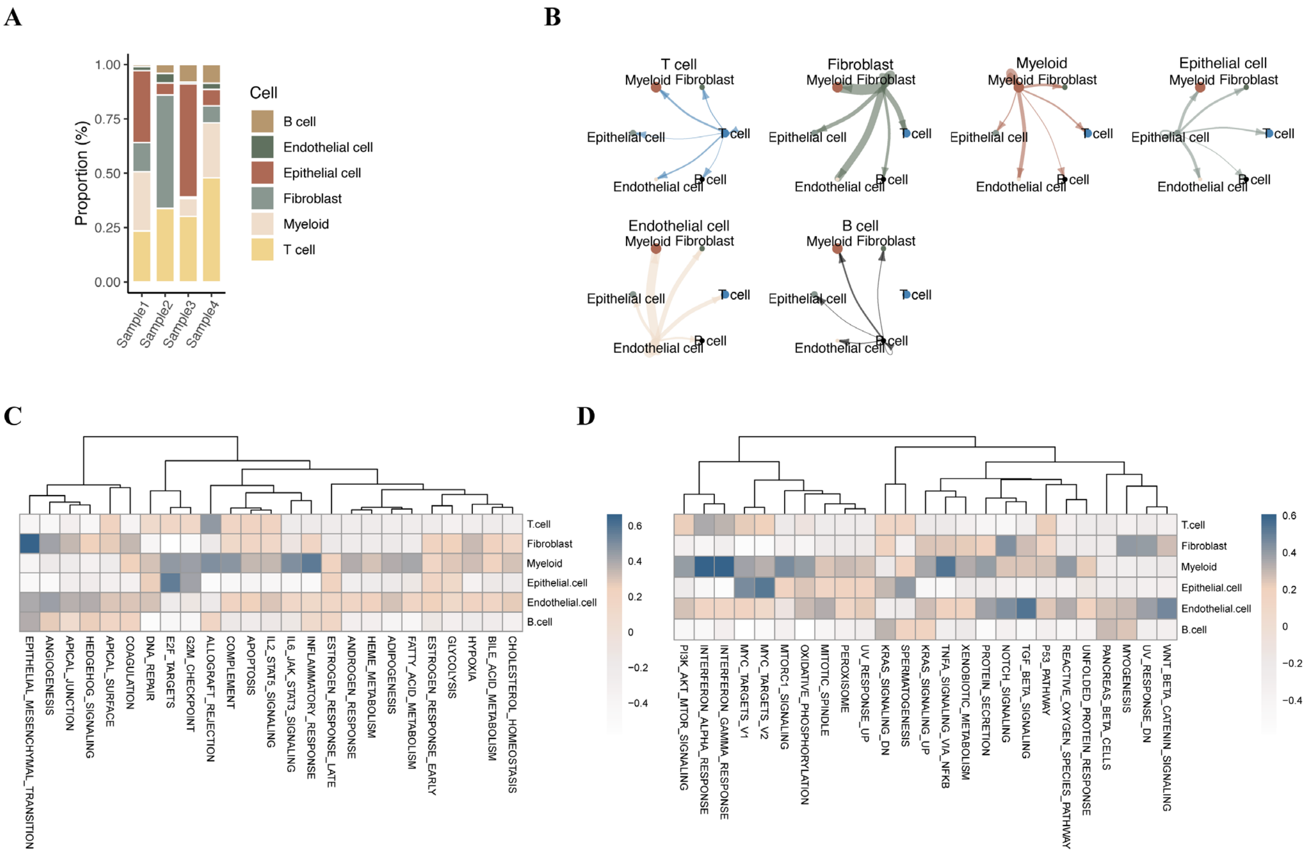

Figure S1. Signaling communication and tumor functional pathway studies of EMT-related cell types

A. Histogram of the cell type share of single cell sequencing data from four SOC patients; B. Cell chat analysis depicting the communication interactions of major SOC cell types, including T cell, Myeloid, Fibroblast, Epithelial Cell, Endothelial cell, and B cell; C. GSVA scores reveal the correlation between the expression of different cell types and major tumour pathways; D. Correlation heat map showing the expression of different SOC cell types and major tumour pathways as reflected by GSVA scores.

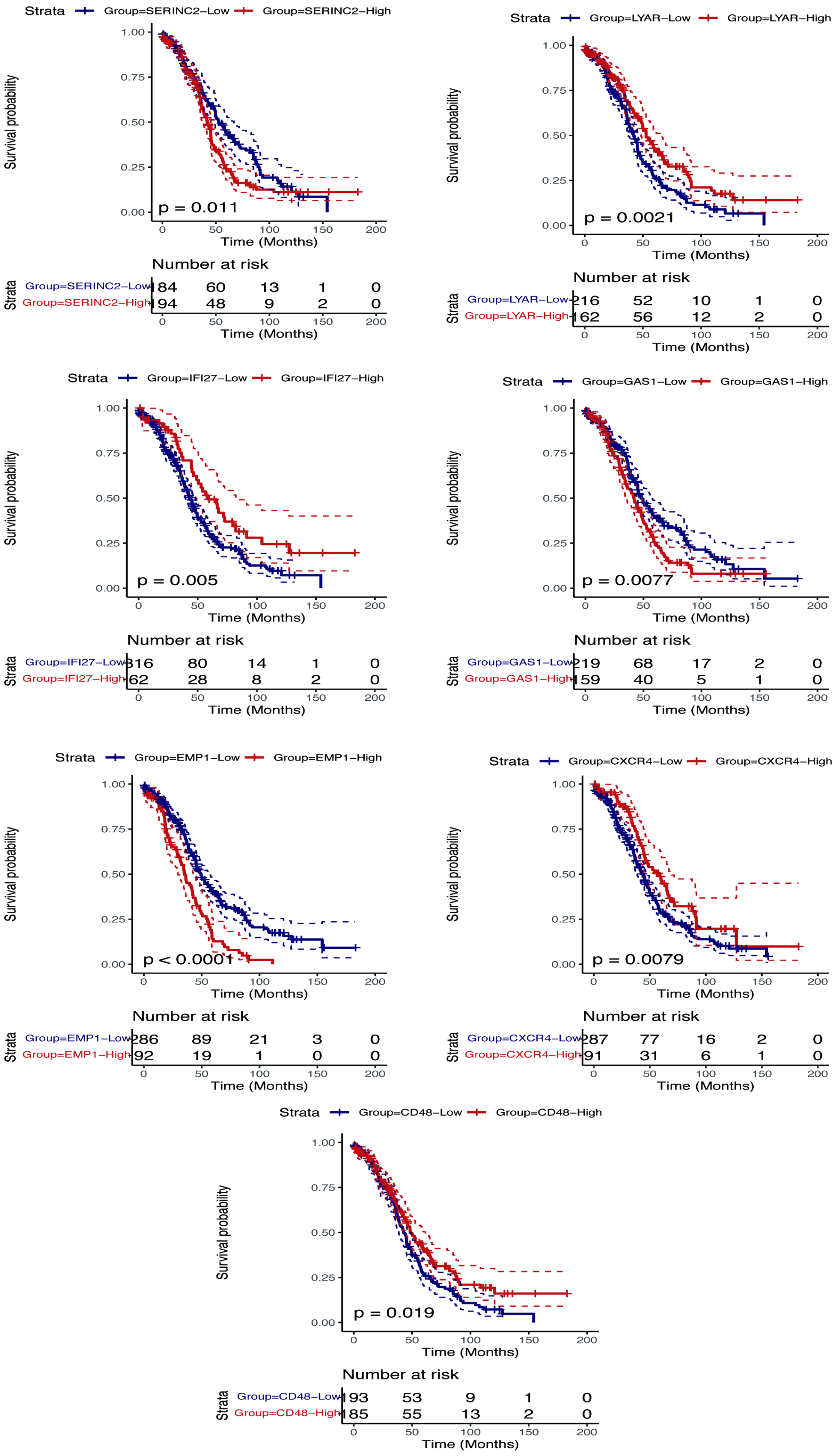

Figure S2. Prognostic properties of model genes

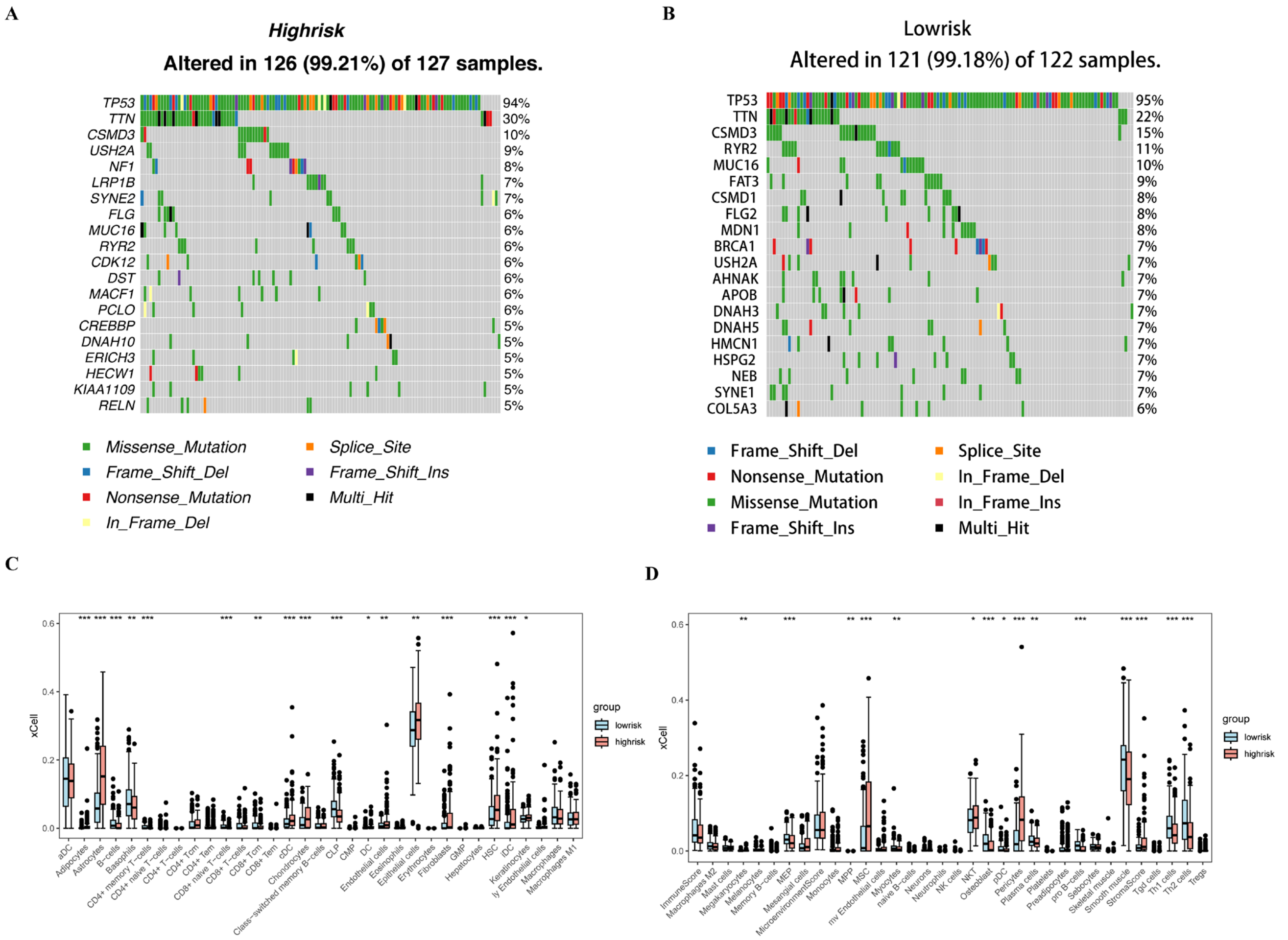

Figure S3. Pathway analysis of EMT-mediated SOC immune infiltration by genetic analysis and XCell method

A. Gene mutation exhibition and descriptive analysis in the EMT high-risk group; B. Gene mutation exhibition and descriptive analysis in the EMT low-risk group; C. Box line plot showing the correlation of expression of multiple tumor immune cells with EMT risk scores in the XCell method; D. Box line plot showing the correlation of expression of multiple tumor immune pathways with EMT risk scores in the XCell method.
